# Aminolaevulinic acid-induced photodynamic therapy: cellular responses to glucose starvation

**DOI:** 10.1038/sj.bjc.6600234

**Published:** 2002-04-22

**Authors:** L Wyld, M Tomlinson, M W R Reed, N J Brown

**Affiliations:** Section of Surgical and Anaesthetic Sciences, Division of Clinical Sciences, University of Sheffield, Floor K, Royal Hallamshire Hospital, Sheffield S10 2JF, UK

**Keywords:** photodynamic therapy, aminolaevulinic acid, nutrient deprivation

## Abstract

Photodynamic therapy is a cancer treatment based on the interaction of light, oxygen and a photosensitiser. Protoporphyrin. IX is an endogenous photosensitiser derived from the pro-drug aminolaevulinic acid. Tumours contain areas of hypoxia and hypoglycaemia. Tumour cells adapt to these conditions by stress protein induction which may induce resistance to cancer therapies. The effect of chronic hypoglycaemia on sensitivity to aminolaevulinic acid-induced photodynamic therapy *in vitro* was studied in MCF-7, human breast cancer cells. Following chronic exposure to 0, 1 or 25 mM, glucose, cells were treated with aminolaevulinic acid and the generation of intracellular protoporphyrin. IX measured by spectrofluorimetry. Aminolaevulinic acid-induced photodynamic therapy sensitivity was compared between cells following chronic exposure to 0, 1 or 25 mM glucose. Percentage cell survival was determined by clonogenic assay. Cells cultured in low glucose generated higher levels of protoporphyrin IX compared to standard glucose medium (0 mM glucose: 0.88×10^−5^ ng cell^−1^, 1 mM: 0.86×10^−5^ ng cell^−1^, 25 mM: 0.605×10^−5^ ng cell^−1^, *P*<0.05). However, photodynamic therapy sensitivity was reduced in glucose deprived cells (0 mM glucose: 61% survival, 1 mM: 80.5% and 25 mM: 39.6%, *P*<0.05). Chronic exposure to low glucose induces photodynamic therapy resistance despite increased intracellular concentrations of protoporphyrin IX and may reflect cellular adaptation to chronic glucose deprivation.

*British Journal of Cancer* (2002) **86**, 1343–1347. DOI: 10.1038/sj/bjc/6600234 www.bjcancer.com

© 2002 Cancer Research UK

## 

Photodynamic therapy (PDT), is a cancer treatment based on the interaction of light, oxygen and a photosensitising chemical which results in the generation of cytotoxic reactive oxygen species (Weishaupt *et al*, 1976). Aminolaevulinic acid is the pro-drug for the photosensitiser protoporphyrin IX (PpIX). Photodynamic therapy has certain clinical limitations. Bulky tumours are difficult to treat because of limited light penetration, but also due to biological factors within the tumour. As the tumour mass increases, passive diffusion is no longer adequate to supply metabolic demands (Vaupel *et al*, 1989). Tumours therefore induce their own microcirculation (angiogenesis) but despite this, areas with low levels of oxygen and essential nutrients persist (Gullino *et al*, 1967; Wendling *et al*, 1984). Glucose levels in normal tissues are approximately 5 mM, whereas values for tumours are usually between 0 to 2 mM (Gullino *et al*, 1967). Low tumour glucose levels are exacerbated because tumour cells preferentially metabolise glucose by either aerobic or anaerobic glycolysis, rather than by oxidative phosphorylation (Warberg, 1956). This is thought to be due to gene mutations influencing the expression of the key regulatory enzymes of glycolysis (Vora *et al*, 1985; Hue and Rousseau, 1993; Chesney *et al*, 1999), which results in increased glucose utilisation rates per gram of tissue (Rofe *et al*, 1988; Heacock and Sutherland, 1990).

Tumour cells may have an increased ability to withstand the resulting adverse conditions. For example mutation of the p53 gene allows cells to continue proliferating despite hypoxia (Graeber *et al*, 1996). Although less well studied, there is recent evidence that similar genes in cancer cells may exist which facilitate tolerance to nutrient starvation (Izuishi *et al*, 2000), such as bcl-2 (Lee *et al*, 1997; Shim *et al*, 1998). When a cell is exposed to nutrient starvation, the cellular energy expenditure is down regulated, primarily by a reduction in protein synthesis, decreased ion channel activity and increased utilisation of endogenous triglycerides (Culic *et al*, 1999). The cell induces a variety of stress proteins, including glucose regulated proteins (GRPs), oxygen-regulated proteins (ORPs), and heat shock proteins (HSPs), which have a protective function and increase tolerance to hypoxia, hypoglycaemia and certain anticancer therapies (Gazit *et al*, 1999; Yu *et al*, 1999). Glucose regulated proteins are constitutively expressed at low levels, but on stimulation with a low glucose level for over 24 h, levels may increase by as much as nine-fold (Gazit *et al*, 1999).

Several groups have studied the effect of stress protein induction, on PDT sensitivity, which appears to be photosensitiser dependent. Gomer *et al* (1991), demonstrated that cells treated with the calcium ionophore A23187, an inducer of GRPs, were significantly less sensitive to Photofrin-PDT. In direct contrast to this, Morgan *et al* (1998), using the same GRP-inducer found PDT sensitivity increased when using Victoria Blue BO (VBBO) as the photosensitiser. The differences were attributed to the intracellular localisation of the two sensitisers, with VBBO mainly localised in the mitochondria and Photofrin II in multiple organelles. However, calcium ionophores have additional cellular effects with innate cytotoxicity and influences on calcium levels within the cell. This may influence the effect of PDT directly (Penning *et al*, 1992). There have been no published studies of the effect of GRP induction on ALA-induced PDT.

The direct effect of glucose deprivation on ALA-induced PDT has not been studied. This might be expected to elicit more complex effects on the cell than simple induction of GRPs. On exposure to glucose deprivation cells enter a phase of reduced proliferation (Mazurek *et al*, 1998), and decreased energy expenditure, with reduction in biosynthetic processes, as well as stress protein induction. In extreme glucose starvation, direct cytotoxicity may be observed. It is thought that under such circumstances, there is an increased production of pro-oxidants by the mitochondria, which exceeds the capacity of the free radical scavenging mechanisms, resulting in cell death (Lee *et al*, 1998). Thus cytotoxicity induced by extreme glucose starvation, acts in a similar way to PDT and may therefore potentiate efficacy.

The effect of increased glucose concentrations on the efficacy and uptake of haematoporphyrin derivative on tumour cells has been studied previously (Thomas and Girotti, 1989). Increasing serum glucose levels enhanced the uptake of haematoporphyrin derivative and cell killing by PDT, an effect attributed to the decreased pH induced by the lactic acid generation as a consequence of increased glycolysis. How such effects influence PpIX generation may be predicted from previous *in vitro* work (Fuchs *et al*, 1997; Wyld *et al*, 1998), suggesting that PpIX generation is optimal between pH 7 and 7.5, decreasing at lower pH values.

Therefore the present study aimed to investigate the effects of chronic glucose deprivation on ALA-induced photodynamic therapy, the rate of intracellular PpIX generation, extra-cellular PpIX accumulation and PDT sensitivity, using the human breast cancer cell line MCF-7. MCF-7 breast cancer cells were selected for this work due to their known sensitivity to glucose deprivation (Yamamoto *et al*, 1999), and the use of clinical PDT in the treatment of cutaneous breast cancer metastases (Mang *et al*, 1998).

## MATERIALS AND METHODS

### Cell lines

#### MCF 7. Human Mammary Carcinoma

This cell line was obtained from the European collection of Animal Cell Cultures (ECACC, Porton Down, UK; Soule *et al*, 1973).

### Cell culture techniques

Cells were cultured in Dulbecco's Modified Eagle's Medium (DMEM, Gibco, UK) supplemented with 10% foetal calf serum (Gibco, UK) and 1% penicillin and streptomycin solution (Gibco, UK). Cells were maintained as exponentially growing monolayer cultures at 37°C in air supplemented with 5% carbon dioxide. Cells were fed every 48 h and passaged once or twice weekly. Cell cultures for passage were detached with trypsin 0.05% and EDTA 0.02% (Gibco, UK).

Standard culture medium contained 25 mM glucose. For chronic glucose deprivation 0 and 1 mM glucose medium was used. In all experimental media, dialysed foetal calf serum was used (Gibco, UK).

### Variation in cell viability and proliferation rates with glucose concentration

Glucose concentrations which caused inhibition of basic metabolic processes but which were not toxic to the cells were required for this study. Low glucose concentrations in the range of 5 mM and below cause induction of glucose regulated proteins after a 24 h incubation (Mote *et al*, 1998). The normal glucose concentration in culture medium for this cell line was 25 mM. Three glucose concentrations were therefore studied: 25, 1 and 0 mM. The rate of cellular proliferation and cell death was studied by culturing cells in these media for up to 10 days by serial cell counting and trypan blue staining. Growth curves were then plotted. Cells were plated at three plating densities into the wells of a 24-well plate (1×10^3^, 5×10^3^ and 1×10^4^ cells ml^−1^). The cells were allowed to attach for 24 h and the medium then replaced with one of the three glucose media. A baseline cell count was performed by trypsinising the cells in a well, staining with trypan blue and counting using a Modified Neubauer Haemocytometer. At 24 h intervals cells were harvested and counted. The cell number was then plotted against time from plating to derive a cell growth curve. At all time points three triplicate repeats for each glucose concentration were studied.

### Protoporphyrin IX determination

Cells were plated into 25 cm^2^ tissue culture flasks and allowed to attach for 24 h. On the basis of the cell counting experiments, plating density was modified for different glucose media to give similar cell densities at the time of assay. The standard culture medium was then removed and replaced with, either: 25, 1 or 0 mM glucose medium. The cells were then incubated for 48 h. The old medium was then removed and replaced with, either: 25, 1 or 0 mM glucose medium to which 1 mM ALA had been added and the cells incubated in the dark for 4 h. The cells were then washed and trypsinised and counted using a Neubauer Haemocytometer and trypan blue to determine the number of viable cells per flask. The concentration of PpIX in the medium was determined at the end of the incubation period by mixing the medium with an equal volume of methanol and perchloric acid. The protein precipitate was removed by centrifugation and the fluorescence of the supernatent determined on a spectrofluorimeter with excitation at 406 nm and emission at 604 nm (Perkin Elmer Ltd. UK). The concentration of PpIX in the medium was then determined by comparison with a standard curve of fluorescence against PpIX concentration. This amount was then adjusted to reflect the amount per viable cell.

The amount of intracellular PpIX was determined by first homogenising the cell pellet using an UltraTurrax tissue homogeniser (Janke and Kunkel, Germany), to disrupt the cell membranes. The cell suspension was then mixed with methanol and perchloric acid and the fluorescence of the resulting solution was read on the spectrofluorimeter as above. Again values were corrected per viable cell. For all experiments six triplicate repeats were performed (*N*=18).

### Photodynamic therapy sensitivity-clonogenicity assay

Cells were plated into 96-well plates at a cell density of 2–4×10^4^ cells ml^−1^ and allowed to attach overnight. On the basis of the cell counting experiments, plating density was modified for different glucose medium to give similar cell densities at the time of assay. The cells were then washed and incubated with medium containing 25, 1 or 0 mM glucose for 48 h. One mM ALA was added to the cells in fresh medium of the same glucose concentration and incubated for 4 h in the dark at 37°C. The cells were then exposed to violet light (350 to 450 nm, 86.5 mW cm^−2^), at a light dose of 3 J cm^−2^. (This dose was selected from preliminary experiments which caused approximately 50% cell death response (LD 50) in cells cultured in normal glucose medium (25 mM). The medium was then replaced with standard complete medium without ALA, and the cells returned to the incubator for 24 h. The cells were then trypsinised and re-plated into the wells of 6-well plates and incubated for a further 4 days. At the end of this period the cells were stained with trypan blue and examined under a microscope and the number of viable colonies of more than eight cells were counted in five randomly selected high power fields per well. The mean of this value was taken. Three triplicate repeats were performed for each glucose concentration in addition to the relevant controls for light alone, ALA alone and neither light or ALA. Percentage cell survival was calculated relative to the control cultured with the same glucose concentration.

### Statistical analysis

Comparisons were with an initial analysis of variance (ANOVA, Kruskall Wallace) followed by a Mann–Whitney *U*-Test. Statistical significance was accepted if *P*<0.05. All data are represented as the mean plus standard error of the mean (s.e.m).

## RESULTS

### Cell growth and viability

Following plating there was an initial slight reduction in cell numbers at all plating densities.

**Figure 1:**
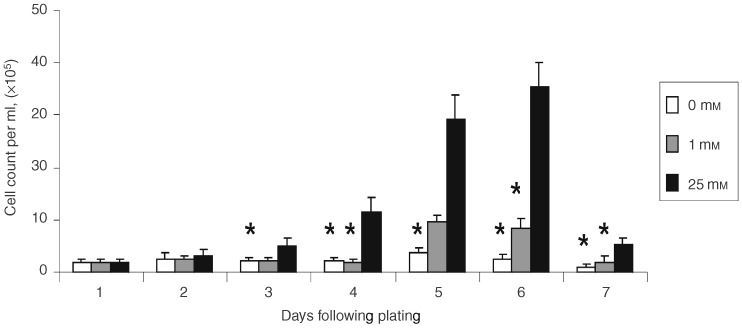
Graph showing cell growth curves for MCF-7 cells cultured in different glucose medium. Mean number of cells per well at different time intervals after plating. Data shown represent means plus standard error of mean. *n*=9. *Denotes statistical significance compared to 25 mM glucose cultures, *P* <0.05, Mann–Whitney *U-*test.

Cells cultured in 25 mM glucose commenced active proliferation within 48 h of plating which reached a maximum at day 6, followed by a sudden decrease in cell numbers. This was accompanied by a visible change in the pH of the culture medium, suggesting a build up of lactic acid. For cells cultured under reduced glucose concentrations, 0 and 1 mM, the increase in cell numbers was delayed and peaked at day 5 at a significantly lower level than in the 25 mM glucose medium (day 5 mean cell counts: 0 mM glucose: 3.83×10^5^ cells ml^−1^, 1 mM glucose: 9.62×10^5^ cells ml^−1^ and 25 mM glucose: 29.26×10^5^ cells ml^−1^, *P*<0.05, Figure 1

). After day 5 there was a reduction in cell numbers but unlike in the high glucose medium, this was not accompanied by a decrease in the pH of the medium (Figure 1).

### Protoporphyrin IX levels following ALA incubation with various glucose concentrations

#### Total PpIX generation

**Figure 2:**
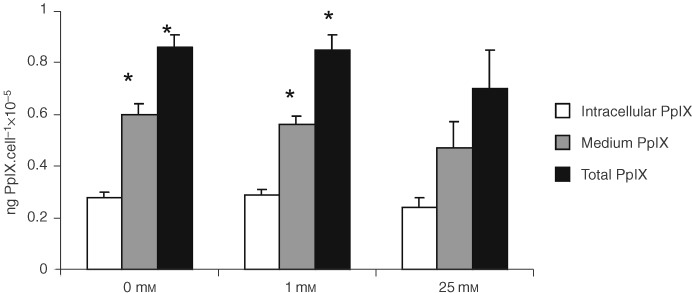
Graph showing PpIX levels following incubation of cells with 1 mM ALA after chronic exposure to varying glucose concentrations. Data shown represent mean plus standard error of mean. *n*=18. *Denotes statistical significance compared to 25 mM glucose, *P*<0.05, Mann–Whitney *U-*test.

Cells grown under conditions of chronic glucose deprivation (0 and 1 mM glucose) produced significantly more PpIX than those grown under normal conditions (*P*<0.05, Figure 2

).

#### Intracellular PpIX levels

Levels of intracellular PpIX were lowest in the cells grown under normal tissue culture glucose concentrations, highest in 1 mM glucose, although the differences failed to reach statistical significance (Figure 2).

#### Extracellular PpIX levels

Cells grown under conditions of chronic glucose deprivation produced significantly more extracellular PpIX than those grown under normal conditions (*P*<0.05, Figure 2).

#### Photodynamic therapy sensitivity

**Figure 3:**
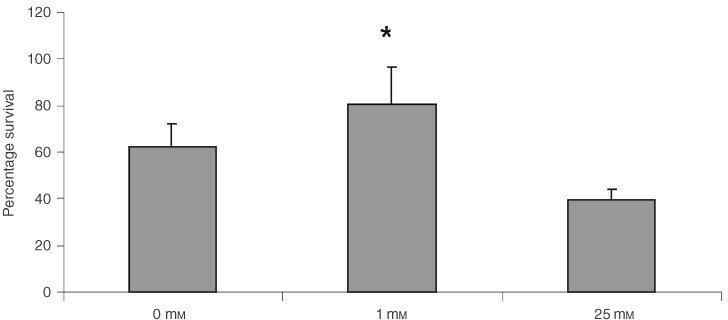
Graph showing percentage survival following PDT after incubation at different glucose concentrations compared to untreated controls. Data represent mean plus standard error of mean. *N*=9. *Represents statistical significance compared to the value at 25 mM glucose, *P*<0.05, Mann–Whitney *U*-test.

Following PDT, cells that were treated under normal glucose conditions were significantly more sensitive to ALA-induced PDT than cells treated under low glucose conditions (0 and 1 mM glucose). The least sensitive cells were those treated at 1 mM glucose, with cell exposed to 0 mM glucose being intermediate in sensitivity (*P*<0.05, Figure 3

). There was no detectable toxicity in controls for ALA alone or light alone treated controls.

## DISCUSSION

This study has demonstrated for the first time that chronic exposure to low glucose concentrations, at levels likely to be found *in vivo* (Gullino *et al*, 1967), inhibits the effect of ALA-induced PDT *in vitro*. The inhibition of response is most pronounced at 1 mM glucose where cell cytotoxicity due to the direct effects of low glucose concentration is not apparent. At zero mM glucose, cytotoxicity due to PDT is increased when compared to cells incubated with 1 mM glucose, but this is not as dramatic as the response of cells in normal glucose medium. These data may suggest that the increased reactive oxygen species generated by glucose deprivation itself (Lee *et al*, 1998), may be potentiating the damage caused by PDT which is also due to the production of reactive oxygen species (Weishaupt *et al*, 1976). This may be partially overcoming the protective effects of GRP induction caused by total glucose deprivation.

The underlying reason for the reduction in PDT toxicity with low glucose concentrations cannot be attributed to changes in the intracellular concentration of PpIX, which has been previously demonstrated to influence PDT sensitivity (Wyld *et al*, 1997), as levels were lowest in the most sensitive cells, i.e. those treated under normal glucose concentrations. The differences in intracellular PpIX generation were small and did not reach statistical significance. The rate at which the cells generated PpIX (total production, i.e. intracellular and medium), was actually increased by chronic glucose deprivation, suggesting either induction of the rate limiting enzyme, thought to be porphobilinogen deaminase (Gibson *et al*, 1998), or a reduction in the rate of conjugation to iron to form haem by the enzyme ferrochelatase. There was a significant increase in the amount of PpIX in the medium, which may reflect increased generation, but it is also possible that the rate of PpIX leakage from the cells was increased. Cells exposed to nutrient deprivation tend to conserve energy by reducing the activity of their ion channels (Culic *et al*, 1999), which may affect the cells ability to retain intracellular molecules.

The effect of an acute reduction in glucose concentration on PpIX generation from ALA has been studied previously and is bi-phasic with maximal PpIX production at 2 mM, decreasing at both higher (4–8 mM) and lower (0 mM) glucose concentrations (Dietel *et al*, 1996). This study exposed the cells to the low glucose concentration for up to 8 h, during which period the ALA was also applied. It is unlikely that this period of time is long enough to induce adaptive changes in the cells such as stress protein induction. This study only looked at total PpIX generated, with no differentiation between intra and extracellular levels and PDT sensitivity was not studied (Dietel *et al*, 1996).

The changes in the rate of PpIX generation cannot be attributed to differences in cell density between the different glucose media. Cell density is known to affect the rate of PpIX generation. Increasing tumour cell density by a factor of 10 increases PpIX production (Moan *et al*, 1998). The present results show a reverse trend in PpIX production despite the higher density of cells in the high glucose cells (30×10^5^ cells ml^−1^ at 0 mM glucose vs 44×10^5^ cells ml^−1^ at 25 mM glucose), suggesting the involvement of additional processes.

The pH of the culture medium is unlikely to have contributed to the differences in PpIX levels, as the medium was replaced at the start of the 4 h ALA incubation period, and the lower pH value would be expected in the most acidic flasks (Fuchs *et al*, 1997), i.e. those with the highest glucose concentrations. The data show the reverse trend.

These findings are of concern for clinical PDT. Those tumour cells exposed to extreme nutrient deprivation often combined with marked hypoxia for prolonged periods are usually not viable and undergo spontaneous cell death. However, cells in less extreme conditions of nutrient deprivation are likely to develop tolerance to the effect of PDT, due to GRP induction. These nutritionally deprived cells are known to have an increased metastatic potential (Schlappack *et al*, 1991), and may therefore serve as resistant foci for tumour progression. Unlike the problem of hypoxic inhibition of PDT (Mitchell *et al*, 1985; Wyld *et al*, 1998), there is a potential solution; infusions of glucose may elevate tumour glucose levels appreciably and therefore reduce the proportion of hypoglycaemic cells. The effect of glucose infusions on Photofrin-PDT have been studied previously and hyperglycaemia enhanced the uptake of Photofrin by the target tissue and increased PDT sensitivity (Thomas and Girotti, 1989). The reason for this effect was suggested to be due to the decrease in pH, which accompanied excess glucose due to its conversion to lactate.

The effect of different glucose concentrations on cellular proliferation rates was predictable. MCF-7 cells are known to be relatively intolerant of low glucose levels, and at concentrations of 5 mM and below, undergo a significant reduction in proliferation (Yamamoto *et al*, 1999). In the current study the effect at zero glucose concentration after 6 days demonstrates that these cells are unable to survive long term without glucose. After 48 h in low glucose concentrations, the added insult of PDT induces cell death, demonstrating that the protective effect of chronic adaptation to glucose deprivation is overwhelmed.

In summary therefore, this study presents the first report of the effect of chronic glucose deprivation on the sensitivity of breast cancer cells to photodynamic therapy. Chronic exposure to low glucose levels has a protective effect on the cells, possibly due to induction of stress proteins, but this requires further study. This may inhibit the efficacy of ALA-PDT in the treatment of bulky tumours and further work is needed to assess whether induction of transient hyperglycaemia may reverse this. It must also be remembered that low glucose and hypoxia usually co-exist and as both have an inhibitory effect, this suggests that PDT for bulky tumours may have inherent limitations.
